# Prodromal services for at-risk youth and their integration with existing programs: A “modular integration” model

**DOI:** 10.1192/j.eurpsy.2021.179

**Published:** 2021-08-13

**Authors:** A. Savic, D. Ostojic, T. Jendricko, P. Brecic, J. Vukojevic

**Affiliations:** 1 Department Of Diagnostics And Intensive Care, University Psychiatric Hospital Vrapce, Zagreb, Croatia; 2 Department Of Psychotherapy S, University Psychiatric Hospital Vrapče, Zagreb, Croatia; 3 Department For Affective Disorders, University Psychiatric Hospital Vrapče, Zagreb, Croatia

**Keywords:** clinical high-risk, prodromes, psychosis, early intervention

## Abstract

Prodromal symptoms may precede onset of the psychotic disorders by years, and while we have instruments for identification of clinical high-risk (CHR), existing predictive models yield low specificity and fail in successfuly predicting transition to psychosis. This, along with the fact that we see a notable decline in transition rates, and the ever-looming lack of resources, means CHR work is still often relegated to research settings. First-episode psychosis (FEP) unit of our institution offers both inpatient and outpatient programs, and while it is common having CHR services emerge from FEP frameworks, there is an inherent risk in drowning the diverse CHR population into very specific FEP interventions and settings, risking additional burden of stigma. Model our institution introduced includes: ⇒Non-dependence on research funding; ⇒Flexible integration in available resources (e.g., FEP interventions, anxiety disorders interventions, substance-use programs); ⇒Dimensional approach with interdisciplinary assessment of capacities/needs non-reliant on supposed diagnosis; ⇒Embedded coordinating clinician ensuring “coordination” of CHR population “dispersed” among different programs; ⇒Collaboration with child psychiatrists allowing smoother transfer of at-risk youth; ⇒Recruitment through sensitized general practitioners and counseling services. No model of care, especially in area lacking conclusive predictive models, can be generalized to different health care systems, as practices and resources vary significantly. Nevertheless, the model presented makes a contribution to the care of CHR population, utilizing/integrating available resources, focusing on dimensional formulation of needs, and avoiding possible stigmatization. Furthermore, widely available CHR services, congruent with any research initiatives, might help us reach the necessary critical mass of data and experience needed for the final push towards clearer prediction models and treatment algorithms.

**Disclosure:**

No significant relationships.

